# Using data from patient registries to answer important research questions

**DOI:** 10.36416/1806-3756/e20240216

**Published:** 2024-07-29

**Authors:** Samia Rached, Cecilia M Patino, Juliana Ferreira

**Affiliations:** 1. Methods in Epidemiologic, Clinical, and Operations Research-MECOR-program, American Thoracic Society/Asociación Latinoamericana del Tórax, Montevideo, Uruguay.; 2. Divisão de Pneumologia, Instituto do Coração, Hospital das Clínicas, Faculdade de Medicina, Universidade de São Paulo, São Paulo (SP) Brasil.; 3. Department of Preventive Medicine, Keck School of Medicine, University of Southern California, Los Angeles (CA) USA.

## PRACTICAL SCENARIO

A group of pediatricians and pulmonologists from the largest referral centers for cystic fibrosis (CF) in Brazil has questioned whether there are genetic, clinical, and treatment distinctions among the different regions of the country. They choose to create a patient registry to compile the characteristics of CF patients in the various regions of Brazil. A few years later, some of these investigators used data from the registry to study the most common types of mutations leading to CF in Brazil and regional differences regarding accessibility to genetic testing.^1^

## WHAT ARE PATIENT REGISTRIES?

A patient registry is an organized data system in which data is systematically collected over time for a population with a specific disease, condition, or outcome.

Patient registries include real world data, potentially providing a realistic image of the target population’s characteristics, treatments, and outcomes. Data are collected periodically and in a timeline that can be used to study the natural course of diseases and changes in the patterns of care over time. Reports of aggregate data are created and made widely available to the public and medical community. Patient registries are especially helpful in evaluating rare diseases because they typically include data from patients at different medical centers and in several regions of a country or continent. Comparing data from different regions may reveal disparities in the severity of disease at the time of diagnosis, the treatments most often used, and short and long-term outcomes, which could inform the development of diagnosis and treatment guidelines, as well as public health policy to improve health equity and patient outcomes. Registries may also be useful for monitoring the real world impact and safety of new treatments, because they include data from high-risk patients, who are usually excluded from randomized controlled trials.^2^

## REGISTRY-BASED STUDIES

Although registries are created mostly to inform clinical management, the accumulated data can be used in order to answer research questions. A registry-based study is an observational method to answer a research question using data from patient registries. Researchers can answer research questions using a patient registry to quickly access data provided by hundreds, or even thousands, of similar patients. In our practical scenario example, the researchers found that only 67% of patients in the Brazilian registry had access to genotyping tests, and that access to newborn screening and age at diagnosis were mediators of the effect that region had on a positive genotyping result.^1^ Multiple studies may be performed by using a single patient registry. Although studies using patient registries are typically retrospective in nature, they have the advantage of including very broad study populations, thus providing results that are more frequently generalizable to the target population than are those of highly controlled randomized clinical trials. The differences between a patient registry and a registry-based study are shown in Table 1.

**Table 1:** Differences between a patient registry and a registry-based study.

Aspect	Patient registry	Registry-based study
Nature	Data collection system, mostly to increase awareness and inform public policy	Designed to answer a focused research question, applicable to the target population of the registry
Follow-up	Long-term, open-ended	Defined by the study objectives
Patient enrolment	All patients within the purpose of the registry (all patients with that condition in the specified area)	Defined by the study objectives and target population
Data collection	Wide range	Restricted to variables needed by the research question
Analysis plan	Routine periodic data analysis	Planned in the study protocol
Data quality	Applied routinely to all data and processes	Study-specific data quality management potentially needed

## CHALLENGES OF PATIENT REGISTRIES

Developing and implementing a registry is not an easy task. It requires human and financial resources to design and implement the project. Case finding and data collection are time consuming and demand adequate training and motivation of stakeholders, to ensure participation and the collection of reliable data. Those can be barriers, and financial and nonfinancial incentives or credits may be used. Sustainability of the project, with continued funding and data collection, also needs to be considered. Registries also need appropriate ethical and legal analysis, including participant consent and strategies to ensure data confidentiality and security. Technological developments may facilitate the design and maintenance of a reliable registry, as well as its use for research and quality improvement projects.^2^

## KEY MESSAGES

Patient registries are a useful source of real-world patient data. Registry-based studies use these databases to answer research questions. 

Developing a patient registry requires human and financial resources. To acquire reliable data, it is essential to have a prepared and motivated team.
